# Transcriptomic Responses of Wheat Anthers to Drought Stress and Antitranspirants

**DOI:** 10.3390/plants14172633

**Published:** 2025-08-24

**Authors:** Misbah Sehar, Philippa Borrill, Laura Vickers, Peter S. Kettlewell

**Affiliations:** 1 Centre for Crop and Environmental Science, Harper Adams University, Newport, Shropshire TF10 8NB, UK; lvickers@harper-adams.ac.uk; 2 Department of Crop Genetics, John Innes Centre, Norwich Research Park, Norwich NR4 7UH, UK; philippa.borrill@jic.ac.uk

**Keywords:** bread wheat (*Triticum aestivum*), vapor gard, abscisic acid, water deficit, gene expression, early meiosis, anthers, antitranspirants, drought stress

## Abstract

Drought severely impacts crop yields, especially wheat. Antitranspirants, which reduce water loss, have been shown to improve crop yield under drought, possibly by increasing pollen viability. To understand the mechanisms, transcriptomic responses were studied in early meiotic wheat anthers extracted from polytunnel-grown plants: well-watered, droughted unsprayed, and droughted plants treated with antitranspirants. Film (Vapor Gard (VG), di-1-*p*-menthene) and metabolic (Abscisic Acid (ABA), 20% S-ABA) antitranspirants were applied at the flag leaf stage (GS39). Well-watered (WW) plant anthers had more upregulated genes (626 genes) than downregulated ones (226 genes) when compared to droughted unsprayed ones. Most of the differentially expressed genes (DEGs) were transcriptionally downregulated (3959 genes) in droughted, treated-plant anthers (ABA and VG) compared with unsprayed (US), and the number of genes with upregulated expression was lower (830 genes). VG-treated plant anthers had more downregulated genes (3325 genes) than ABA-treated ones (634 genes). Carbohydrate or sugar metabolism and related processes were affected in antitranspirant-treated plant anthers with significant downregulation of genes compared to droughted unsprayed ones; in contrast, these processes were upregulated in well-watered anthers, suggesting broad differences in the transcriptional response. However, antitranspirants did not significantly affect pollen viability or yield in treated plants compared to unsprayed plants, suggesting that anthers are more sensitive at the transcriptomic level than subsequent physiological processes determining yield.

## 1. Introduction

Wheat is one of the main staple food crops grown around the globe and extensively researched in terms of abiotic and biotic stresses. Among the abiotic stresses, drought is one of the main factors responsible for reducing yield, thus creating a global threat to food security. If the proper mitigation steps are not taken, either to improve the crop to cope with these severe climatic conditions or to mitigate these climatic changes, 60% of the area under wheat will face severe drought by the end of this century [1]. Selection and breeding of drought-tolerant varieties, introgression of suitable alleles from wild relatives into commercial cultivars, soil, water, and crop management practices are some of the top priorities in regions where drought is predicted to become severe [2,3,4]. Using antitranspirants to reduce water loss from plants under limited water environments is an alternative way to address the problem of drought stress. In simple terms, antitranspirants mainly work by reducing transpiration from plant surfaces. These are the chemical compounds that help to conserve the water status of plants and are categorised as film-forming, stomatal-closing, reflectance-type, and even growth retardants depending on their mode of action [5,6]. Previous studies on antitranspirants have shown increased wheat crop yield by reducing water loss from plants under water stress conditions [7,8,9,10]. The application of antitranspirants at specific growth stages that are sensitive to drought is also critical, as they may not improve yield when applied at less sensitive growth stages [7]. Treatment of the film antitranspirant (Vapor Gard) before the start of meiosis of droughted wheat has the potential to improve crop yield, which might be linked with improved pollen viability [7,11]. Similarly, exogenous ABA application at the flag leaf emergence of droughted wheat under progressive drought and multiple applications of exogenous ABA at four more stages up to anthesis have also indicated improved grain yield under controlled drought [9]. The use of antitranspirants has also been investigated in many cereal and horticultural crops and can be beneficial in the improvement of several other physiological and quality characteristics, along with yield [12]. Therefore, the application of antitranspirants could be one of the ways to improve crop production in water-deficient environments.

Drought tolerance mechanisms also vary in different crops and varieties depending on their gene expression patterns, which might later influence their yield. For example, a study on two wheat varieties with different drought tolerance mechanisms indicated that the variety with higher yield and elevated flag leaf ABA content showed more changes at the transcriptomic level than the other with lower crop yield [13]. Therefore, it was concluded that the differential role of ABA in plants during the grain-filling stage may regulate the major changes at the molecular and physiological levels that can lead to higher yield and superior adaptation of a variety under water deficit conditions. Another transcriptomic study [14] on wheat grown in rain shelters revealed that the early reproductive stages (spike differentiation stages) of wheat are more sensitive to drought and can affect crop development, gene expression patterns, and crop yield more than drought stress during the flowering stage. Additionally, some of the drought-responsive differentially expressed genes (DEGs) were found, with links to photosynthesis, stomatal movement, and floral developmental processes.

Drought stress during the reproductive stage of anthers is the main cause of pollen sterility in many crops, as it affects the processes related to carbohydrate availability, metabolism and distribution, and hormonal signalling and pathways and alters their gene expression patterns to cope with stress environments [15]. Reduced pollen fertility from the reproductive stage water stress is the main cause of grain loss, and the key to maintaining pollen fertility and grain number might be associated with sink strength and carbohydrate supply to anthers under drought stress [16]. In rice, male fertility is drastically affected by abnormal starch accumulation in anthers, and mainly genes involved in microspore/tapetum development, formation of the cell wall, and starch synthesis are affected [17]. Defects or disruption of sugar metabolism during anther and pollen development in crop plants often lead to male sterility [18]. In wheat, it has been proposed that the downregulation of *IVR1* and *IVR5* invertase genes in wheat anthers under water stress might cause reproductive failure and lead to pollen sterility [19]. However, no significant difference was observed in the expression level of the *IVR5* gene between film antitranspirants and unsprayed treatments in wheat plant anthers [20]; therefore, it was concluded that there was no evidence that film antitranspirants affect the expression pattern of this drought-sensitive gene to alleviate drought stress and to improve pollen viability.

Although several transcriptomic studies have been carried out on wheat under drought stress and on reproductive parts of various crop plants to understand different tolerance mechanisms, there has been no transcriptomic study in wheat following the use of antitranspirants. Therefore, here, we aimed to understand the effects of antitranspirants at the transcriptomic level in wheat anthers (at the early meiosis stage—leptotene-zygotene) and whether any of these transcriptional changes can be linked to changes at the physiological level, such as pollen viability or crop yield. Also, anther samples for the transcriptomic analysis were taken from polytunnel-grown plants (in the field) to understand the gene expression responses in natural field environmental conditions, rather than a controlled glasshouse or growth chamber environment, as is mostly performed in various previous transcriptomic studies related to drought stress. Differentially expressed genes from droughted antitranspirant-treated (ABA and VG) plant anthers and well-watered anthers, compared with droughted unsprayed ones, were investigated for their biological functions. Moreover, DEGs related to invertase genes, starch and sucrose synthesis genes were explored to understand their expression patterns in treated-plant anther samples under drought stress and well-watered samples, as these play a crucial role in carbohydrate or sugar metabolism in anthers with links to anther/pollen development or pollen viability in various literature studies.

## 2. Materials and Methods

### 2.1. Experimental Site, Design, and Treatments

Spring wheat variety (Chilham) was sown in a randomised complete block design in four polytunnels (rain shelters) in the Flatt Nook Field of Harper Adams University, Shropshire, UK (52°46′ N, 2°25′ W), in 2022. The soil at the site is loamy sand with a field capacity (FC) of 22% volumetric water content (VWC) and a permanent wilting point of 8% VWC [10].

There were eight experimental plots in each polytunnel: six droughted plots and two well-watered plots, with sizes of 1 × 1 m each. Well-watered plots were not randomised, as they were present at the front corner of each polytunnel to enable watering by drip irrigation (five 1 m drip tapes with dripper spacing 20 cm and output of 250 L/ha, TSX 506 T-Tape, Access Irrigation Ltd., Northampton, UK) and to avoid water entering the droughted plots. Therefore, these were not included in the statistical analysis. Watering of the well-watered plots was performed three days a week (Monday, Wednesday, and Friday) for one hour to keep the mean soil moisture value up to 16–18% VWC (approximately 70–80% of FC) in the top 50 cm of the soil profile.

Two types of antitranspirants (Vapor Gard (VG) (96% di-1-*p*-menthene, Miller Chemical and Fertilizer LLC, Hanover, PA, USA) at 1 L/ha and ABA (20% S-Abscisic Acid, ProTone SG Plant Growth Regulator Soluble Granule, Valent Bioscience LLC, Libertyville, IL, USA) at 30 g/ha (containing 6 g/ha of S-ABA)) were applied at the flag leaf stage (GS39) [21] before the start of meiosis in droughted experimental plots. Both VG and ABA were applied with an application rate of 200 L/ha. Spraying of antitranspirants was performed with the help of a handheld sprayer (5 L Hozelock pressure sprayer). Amongst the six droughted plots, two were unsprayed, two were sprayed with VG, and two were sprayed with ABA.

### 2.2. Planting, Agronomic Practices, and Management

Based on the previous crop grown in the polytunnels and the nutrient management guide (RB209) [22], nitrogen fertiliser was broadcast in the polytunnels at the rate of 130 kg N/ha (as ammonium nitrate) before planting. Seeds were sown manually (18 June 2022) at a depth of 2 cm in soil at the rate of 400 seeds per m^2^. There were six rows in each experimental plot of the polytunnel with row spacing of 15 cm. Seedlings emerged after 9–16 days of sowing with water given to each plot 2–3 days a week up to the growth stage of GS31 (first node visible) to help the germination process and also to store enough moisture in the soil so that when progressive drought started after GS31 till physiological maturity, it remained above the permanent wilting point. Weeding was performed manually during the cropping season, and to keep out birds, rabbits, and other animals, the front and back of the polytunnels were covered with netting and rabbit fencing.

### 2.3. Measurements and Sampling

#### 2.3.1. Air Temperature, Relative Humidity, and Soil Moisture

Temperature and relative humidity data inside the polytunnels was recorded daily with Tinytag Ultra 2 data loggers (Gemini Data Loggers Ltd., Chichester, UK). Solar radiation data was taken from the meteorological station based at Harper Adams University (approximately one kilometre away from the location of the research field).

Soil moisture was measured (via access tubes inserted randomly in four droughted plots of each polytunnel and one in a well-watered plot) once a week up to a depth of 50 cm with a time-domain reflectometer (TDR) (TRIME-PICO IPH/T3, IMKO Micromodultechnik GmbH, Ettlingen, Germany). Readings (in the form of percentage volumetric water content) were taken from four depths (20, 30, 40, and 50 cm) from each access tube to calculate the mean soil moisture values in the top 50 cm of the soil profile. Measurements were started three weeks after sowing and continued until physiological maturity.

#### 2.3.2. Collection of Anthers at Leptotene-Zygotene of Meiosis I

For the collection of anthers at the early meiosis stage, wheat stems (main stem or primary tillers, with 3–4 leaves cut from the middle of a plant stem with scissors) were collected around GS41 from four plots (well-watered, unsprayed, ABA-treated, and VG-treated plants) in one of the polytunnels. Four to five stems were collected daily or every other day during the three weeks, depending on the distance between the penultimate leaf and the flag leaf sheath. If the distance between the auricles of the penultimate leaf and the flag leaf sheath was between 4.5 to 6 cm, then anthers could be found in the spike (3–4 cm long) at the leptotene-zygotene stage of meiosis I. Collected stems were taken to the laboratory, where well-watered ones were put in a beaker with water, while droughted ones were placed in a 5% PEG-400 solution. This was performed to induce osmotic stress (with an estimate of its concentration from Janes [23]) during the stem storage until anthers were extracted from spikes. Both beakers with stems were placed in a refrigerator at 4 °C until anthers were extracted between 1 to 9 days from different samples (Table 1). Wheat stems were collected from one of the polytunnels, with replicates taken from only one experimental plot of each treatment to reduce the variability at the transcriptomic level.

Anthers were extracted (using No. 5 fine forceps) from spikes under a dissecting microscope (Zeiss Stemi 305). One anther was taken from one of the spikelets in the middle of a spike (either from the first or second floret) and put on a glass slide, and one drop of 0.5% acetocarmine solution was added and then heated for 5 s over a flame of a Bunsen burner. A coverslip was placed on the anther and gently tapped with the back of a forceps to squash it. The glass slide was placed under a light microscope (Zeiss Primostar 3) under 40x–100x objectives to see if the anther was at the leptotene-zygotene stage or not, and if it was, then the remaining two anthers (from the same floret) were collected from the spikelet and placed in 500 µL RNAlater solution in a 1.5 mL Eppendorf tube. Approximately 50 anthers were collected in one Eppendorf tube for each sample, checking each time by taking one anther from each spikelet to make sure they were at the right stage of meiosis. There were four main anther samples (WW, US, ABA, and VG) with three biological replicates, making a total of 12 samples (Table 1). Each anther sample contained anthers from multiple tillers. Each sample was stored in a refrigerator at 4 °C overnight after collecting 50 anthers in an Eppendorf tube to allow thorough penetration of RNAlater solution into the anthers. The next day, each sample was stored in a −80 °C freezer until further processing.

#### 2.3.3. Pollen Viability

Pollen viability was determined by the presence or absence of pollen starch accumulation via staining of pollen grains. It was assessed according to the method [11] by randomly collecting 10–15 freshly dehisced anthers from the spikes of well-watered, unsprayed and antitranspirants (ABA and VG)-treated plants in Lugol’s solution in dark Eppendorf tubes. After collection from the field, Eppendorf tubes were stored in the dark in a refrigerator at 4 °C, and within a week, counting of viable (darkly stained) and non-viable (partially or unstained) pollen was performed under a light microscope (10x objective, Zeiss Primostar 3) using a Sedgewick Rafter counting chamber. Three replicates of ten random grids were counted for each sample, and the mean percentage of viable pollen was calculated.

#### 2.3.4. Yield and Yield Components

To assess the performance of antitranspirant-treated plants and unsprayed plants in droughted conditions in the polytunnels, yield and yield components were measured. Spike density per m^2^ and thousand-grain weight were calculated according to the method [24], along with the number of grains per spike, number of grains per m^2^, and grain yield. Yield data from the well-watered plots was also collected, but it was not included in the statistical analysis, as these were not randomised in the four polytunnels.

#### 2.3.5. RNA Extraction from Anthers

RNA extraction from collected anthers was performed according to the protocol [25] (using an RNA extraction kit from Zymo Research). However, anthers were used rather than wheat stigmas in the original protocol. Samples were first placed in a refrigerator from −80 °C freezer to defrost, and after RNAlater solution was removed from the samples, 800 µL of TRIzol reagent (phase separation step) was added following the grinding of anthers in a tissue homogeniser. Further steps (to purify the aqueous phase) were followed according to the protocol. Nanodrop was used to assess the quantity and quality of extracted RNA. Samples were sent for sequencing to the company (GENEWIZ UK Ltd., Hope End, Takeley, Essex, CM22 6TA, UK).

### 2.4. Transcriptomic Data Analysis

#### 2.4.1. Pre-Processing of Raw Data Files

Reads (150 bp paired-end reads) were trimmed using a trimmomatic tool to remove the adapters and reads of less than 80 base pairs (bp). Trimmed sample files were pseudoaligned using Kallisto (v0.46.1) to the wheat RefSeqv1.0 annotation v1.1 transcripts [26]. After mapping, tximport (v1.16.1) was used to combine the count and transcript per million (TPM) data of all samples into one data frame.

#### 2.4.2. Differential Expression Analysis

DESeq2 (v1.28.1) [27] was used to compare samples between different conditions. After running DESeq2 on raw count data of all samples and removing low-confidence genes, the count data was transformed using the vst function to create the PCA plot (via the plotPCA function) to visualise the overall effects between different treatments and their replicates. PCA plot indicated that there was high variability within replicates of each treatment; therefore, instead of running DESeq2 across samples from all treatments, a set of three contrasts were compared separately to find the differentially expressed genes. Three different contrasts (well-watered vs. unsprayed (WW/US or WW vs. US), ABA vs. unsprayed (ABA/US or ABA vs. US), and Vapor Gard vs. unsprayed (VG/US or VG vs. US) were analysed in the DESeq2 analysis to find differentially expressed genes in each contrast comparison. For each pair of treatment samples, in contrast, the raw count data was used to perform DESeq2 analysis using unsprayed samples as a reference. Before performing the differential analysis, data of each contrast was filtered to include only high-confidence genes, expressed at >0.5 TPM in contrast samples, and the low-confidence genes with low expression were removed [28]. In the well-watered samples, 55,754 genes, 56,213 genes in unsprayed, 55,868 genes in ABA, and 55,890 genes in VG samples were retained at >0.5 TPM. This process was performed for each contrast separately to find the differentially expressed genes (DEGs) using a *p*-adjusted value of <0.05 and a log2 fold change value of >1 and <−1 to find upregulated and downregulated DEGs, respectively.

#### 2.4.3. GO Enrichment Analysis

For the gene ontology (GO) enrichment analysis, first, GO terms for RefSeqv1.0 were converted to v1.1 annotation [29], by keeping genes that were >99% identical to >90% of the sequence (from v1.0 to v1.1). Then, GOseq (v1.40.0) was used to get the GO enrichment terms for each contrast separately using the upregulated and downregulated genes of each contrast obtained from the differential analysis (padj < 0.05 and log2 fold change >1 or <−1).

#### 2.4.4. Invertase Genes, Starch, and Sucrose Synthesis Genes

Two lists of invertase genes [30,31] were compared with the genes expressed in this study. Also, homoeologs of expressed invertase genes were found using the EnsemblPlants website (https://plants.ensembl.org/Triticum_aestivum (accessed on 15 August 2023), [32]) and grouped to see whether all three homoeologs or one of them was expressed in different anther samples. Lists of starch-related genes from studies [33,34] were also compared with the expressed genes of this study to understand the type of starch-related genes expressed in different anther samples. Moreover, the sucrose biosynthesis gene list [35] and some sucrose synthase genes from the EnsemblPlants database were compared with DEGs of different samples.

#### 2.4.5. Drought Tolerance or Response Genes

To understand the response of drought-related DEGs expressed in different anther samples, some of the drought tolerance/response genes obtained from various literature studies [14,17,36,37,38,39,40,41] or from the EnsemblPlants and wGRN database (http://wheat.cau.edu.cn/wGRN/ (accessed on 1 January 2025), [42]) were compared with the expressed genes. Rice orthologs of some of these genes were found using EnsemblPlants via BioMart or the gene browser tool from wGRN.

### 2.5. Statistical Analysis

For physiological parameters (pollen viability, yield, and yield components), R Studio was used to apply ANOVA with a randomised complete block design for comparing data from different antitranspirant-treated and unsprayed plants, along with the Tukey post-hoc test after ANOVA analysis. Before performing ANOVA, Levene’s test was used to check the homogeneity of variances between different treatments, and the Shapiro-Wilk test was used to check the normality of data. Data from well-watered plants was not included in the statistical analysis.

## 3. Results

### 3.1. Soil Moisture Decreased Substantially in Droughted Plots

**Figure 1:**
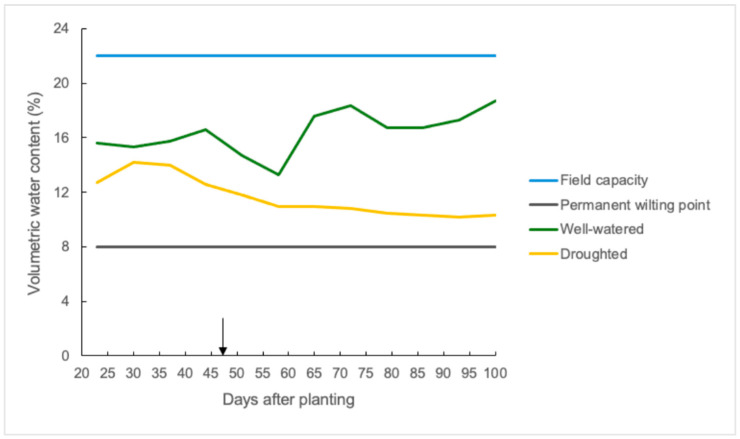
Soil moisture (volumetric water content percentage) in the top 50 cm of the soil profile from the four polytunnels (rain shelters) of the field experiment with values of field capacity and permanent wilting point along with mean soil moisture values of well-watered and droughted plots with antitranspirant treatments (ABA and VG) at the flag leaf stage (GS39) after 47 days of planting as shown in black arrow. Moisture values were taken once a week until plants reached physiological maturity.

Soil moisture in the top 50 cm of soil decreased considerably as days after planting increased in the droughted plots of the polytunnels (Figure 1). The volumetric water content (VWC) of the soil was around 13% (59% of field capacity) when measurements were started 23 days after planting, and it decreased to 10% (45% of FC) by the end of the grain-filling period in the droughted plots. The mean soil moisture value in the well-watered plots was around 17% (77% of FC) during most of the cropping season, except on a few days when it fell between 13 to 15% due to a heatwave at that time.

During the cropping period, the daily mean temperature and relative humidity were around 18 °C and 72%, respectively, inside the polytunnels (Figure S1a,b), with a mean daily solar radiation of 14 MJ/m^2^/day (Figure S1c). There were two heatwaves during the cropping season, with the maximum day temperature exceeding 40 °C on two days in July and for six consecutive days in August 2022 inside the polytunnels (Figure S1a).

### 3.2. Major Transcriptomic Responses from Antitranspirant Treatment Under Drought Stress

Antitranspirants (ABA and VG) were sprayed on droughted plants at GS39, and anthers were harvested for RNA-seq from plants around GS41 (sampling started 10 days after spraying at the early meiosis stage from well-watered and droughted plants, including unsprayed, ABA-treated, and VG-treated) and sequenced. The sequencing data was aligned with the wheat reference genome RefSeqv1.1 annotation using Kallisto. On average, samples had 33 M reads, and 26 M reads were pseudoaligned (80%) using Kallisto (Table S1).

**Figure 2:**
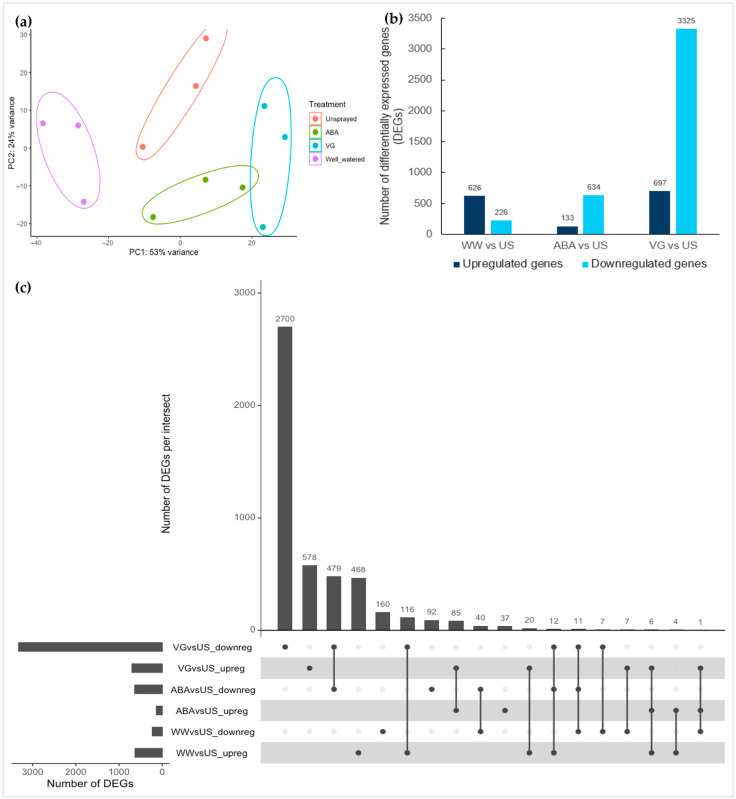
Variation between different anther samples (**a**), differentially expressed genes in different contrast comparisons (**b**), and overlapping genes between different contrasts (**c**). (**a**) Principal component plot of anther samples showing the maximum and second most variation (PC1 and PC2) between different treatments and their replicates. Ellipses are drawn only to show groups of different treatment replicates; (**b**) Number of differentially expressed upregulated and downregulated genes (padj < 0.05, log2 fold change > 1 (upregulated), and < −1 (downregulated) in three types of contrasts comparisons (well-watered vs. unsprayed (WW vs. US), ABA vs. unsprayed (ABA vs. US) and VG vs. unsprayed (VG vs. US))) after DESeq2 analysis; and (**c**) Upregulated and downregulated genes in different contrast comparisons of WW vs. US, ABA vs. US, and VG vs. US samples. The plot shows the number of upregulated and downregulated DEGs that overlap across different contrast samples in intersects. The vertical bars represent the number of either upregulated or downregulated DEGs in each intersect, with the filled circle below showing which contrast sample it belongs to, while the horizontal bars represent the total number of either upregulated or downregulated DEGs for that sample.

Principal component analysis of transformed count data showed the variability between different types of samples and how the replicates of different treatments vary from each other. It can be seen in Figure 2a that replicates are not closely clustered together, which may be due to plant stems and anthers harvested on different dates (Table 1) for each of these replicate samples, which explains the variability between replicates in the PCA plot, or it might be because of their innate biological variability. Therefore, for understanding the transcriptional changes between different treatments, three main types of contrasts (WW/US, ABA/US and VG/US) were compared in DESeq2 separately using unsprayed as a reference.

More than 5000 differentially expressed genes (DEGs) were identified in different contrast comparisons. The number of downregulated genes was lower in the well-watered contrast (WW/US) (226 genes) in comparison to upregulated DEGs (626 genes) (Figure 2b), while contrary to this, the total number of downregulated DEGs in two droughted contrasts (ABA/US and VG/US) were very high (3959 genes) in comparison to upregulated DEGs (830 genes). This suggests that most of the genes involved in different developmental processes in anthers at the early meiosis stage were transcriptionally suppressed with the spraying of antitranspirants under drought stress compared to well-watered anthers. Moreover, in drought contrasts, the number of downregulated DEGs was higher in the VG/US contrast (3325 genes) than in the ABA/US contrast (634 genes), with upregulated 697 genes and 133 genes in each, respectively (Figure 2b, Table S2). This shows that VG (film antitranspirant)-treated plant anthers had a high number of differentially expressed genes, while most of these genes were not differentially expressed in ABA-treated plant anthers, suggesting a unique response in the two types of antitranspirants.

To find the overlapping and unique genes between different contrast comparisons, upregulated and downregulated DEGs were compared (Figure 2c). In two antitranspirant contrasts (ABA/US and VG/US), 85 upregulated genes were common and 479 downregulated genes. Most of the DEGs that were found in ABA anthers were also found in VG anthers for both upregulated and downregulated genes, which suggests that VG induced most of the ABA responses but also had additional effects. There were a few upregulated overlapping genes between WW/US and VG/US contrasts (20 genes) that could suggest a similar response of some genes with film antitranspirant as in well-watered anthers. Moreover, there were some unique genes (upregulated and downregulated DEGs) in both types of antitranspirant-treated contrasts (ABA/US—37 genes and 92 genes and VG/US—578 genes and 2700 genes, respectively), indicating some differences in molecular responses.

### 3.3. GO-Enriched Terms Linked to DEGs

To understand the functions of genes involved in different biological processes, GO enrichment analysis was performed, and various GO terms were enriched in three contrast comparisons (Table S3). In the first contrast (WW/US), the GO terms related to cell cycle (heterochromatin/chromosome organisation and nucleosome assembly), carbohydrate/fructose metabolism, and transport were highly enriched amongst upregulated genes. A few main GO terms related to water stress, such as hydrogen peroxide, heat, reactive oxygen species, protein folding/unfolding, and abscisic acid response, were enriched amongst downregulated genes in the well-watered in comparison to unsprayed droughted samples.

There were very few GO terms enriched for upregulated genes in the second contrast (ABA/US), as the number of upregulated genes was lower in comparison to other contrasts of well-watered and VG samples. Genes enriched for GO terms involved in transcription, respiratory or oxidative burst were the prominent ones. A few main terms enriched for downregulated genes were related to carbohydrate metabolism, oxidation-reduction, flavonoid biosynthesis, photosynthesis, nutrient ion transport, sporopollenin biosynthetic process, and hormones.

In the final contrast comparison of VG and US samples, a few top GO terms associated with upregulated genes were related to water stress, oxidative burst, heat, transcription, hormones, nutrient ion transport, and pollen development. In downregulated genes, the few main GO terms enriched for biological processes involved in photosynthesis, cell cycle, carbohydrate metabolism, oxidation-reduction, protein dephosphorylation, hormones, and nutrient ion transport.

#### Hormonal Responses, Pollen/Anther, Carbohydrate-, or Sugar-Related GO Terms

**Figure 3:**
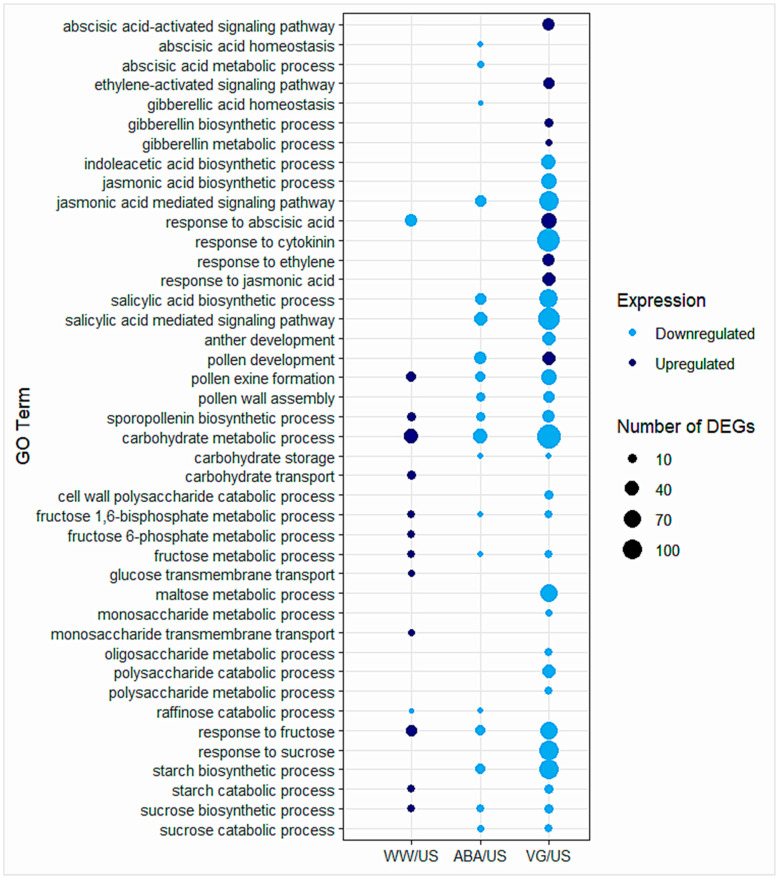
GO terms enriched for biological processes related to hormones, pollen/anther, and carbohydrate- or sugar-related processes in three contrast comparisons of well-watered, ABA, and VG (droughted)-treated plant anthers in comparison to unsprayed. Dark blue represents the GO terms enriched for upregulated genes, and light blue represents the GO terms enriched for downregulated genes. The size of each circle depicts the number of differentially expressed genes (DEGs) enriched for the GO term. More details on GO terms enriched for various biological processes can be found in Tables S3 and S4.

A few GO terms related to hormones were separated to observe gene expression changes in different anther samples (Figure 3, Table S4). Some of the main hormonal GO terms enriched amongst DEGs were related to abscisic acid, gibberellin, jasmonic acid, salicylic acid, ethylene, and cytokinin. Most of the hormonal terms enriched for DEGs were observed in droughted anthers, in contrast to well-watered anthers. In ABA samples, all the hormonal terms were enriched for downregulated genes, while in VG samples, both upregulated and downregulated enriched terms were observed.

GO terms related to pollen exine formation and sporopollenin biosynthetic process indicated a clear difference between well-watered and droughted, treated plant anthers (both ABA and VG), with significant upregulation and downregulation of these processes in each condition, respectively (Figure 3, Table S4). GO term enriched for pollen development showed contrasting expression in droughted ABA anthers (downregulation) and VG anthers (upregulation) when compared with unsprayed, while in well-watered anthers, this GO term was not enriched amongst DEGs. The upregulated DEGs in VG anther samples that were enriched for the pollen development GO term (GO:0009555) are given in Table S5.

Genes related to carbohydrate or sugar metabolism played a significant role in terms of pollen fertility. So, GO terms related to carbohydrate/sugar metabolism and their transport related processes were separated (Figure 3, Table S4), which indicated that in the well-watered plant anthers, genes involved in the carbohydrate/sugar metabolism processes were upregulated, and in droughted conditions (antitranspirants treated), both in ABA and VG plant anthers, genes were downregulated when compared to unsprayed. It also revealed some genes or related GO terms that were not differentially expressed in some samples while expressed in others, suggesting variable responses in different antitranspirant-treated plant samples and well-watered ones.

### 3.4. Downregulation of Invertase, Starch, and Sucrose Synthesis Genes with Antitranspirants

**Figure 4:**
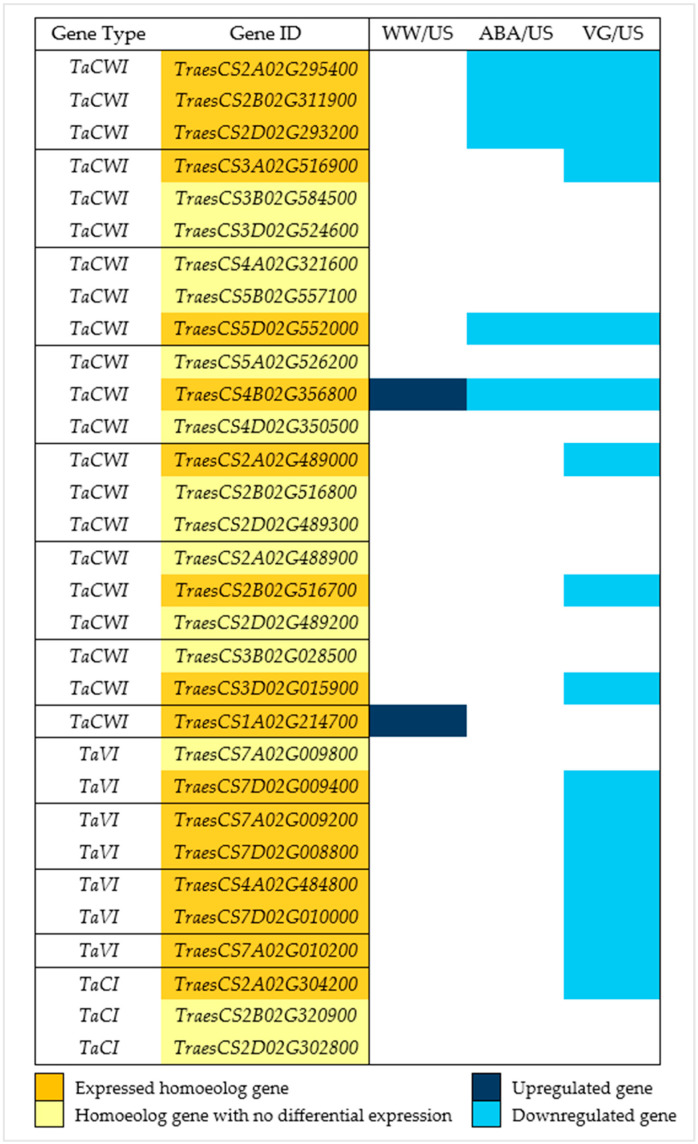
Invertase genes differentially expressed in well-watered, ABA, and VG (droughted)-treated plant anthers compared to unsprayed plant anthers. Dark blue represents upregulated genes, and light blue represents downregulated genes (padj < 0.05, log2 fold change > 1 (upregulated genes), and <−1 (downregulated genes)). Golden yellow represents the expressed homoeologs genes (in different contrast samples) in 13 homoeolog gene groups separated by a solid black line, with depiction of homoeologs genes that were not differentially expressed in light yellow. *TaCWI* represents cell wall invertase genes, *TaVI* represents vacuolar invertase genes, and *TaCI* represents cytoplasmic invertase genes.

Previous studies showed the importance of invertase genes in carbohydrate metabolism in anthers; 17 invertase genes were found with differential expression in various samples (Figure 4). One cell wall invertase gene (*TraesCS4B02G356800*) showed a clear difference between samples, with significant upregulation in WW/US contrast and downregulation in two other droughted contrasts (ABA/US and VG/US), while another cell wall invertase gene (*TraesCS1A02G214700*) expressed only in first contrast (WW/US) with significant upregulation in well-watered anthers, while it was not differentially expressed in droughted anthers. All the other expressed invertase genes were downregulated, with only five genes differentially expressed in the ABA/US contrast comparison, sixteen genes in the VG/US contrast, with five genes common in both. Homoeolog genes were grouped together to see their expression pattern; out of 13 homoeolog gene groups, only 1 homoeolog was differentially expressed in 10 groups, while in the other 3, all the homoeolog genes were expressed. Invertase genes in eight of these homoeolog groups belonged to the cell wall invertase gene family (*TaCWI*, invertase genes expressed in the cell wall), four belonged to the vacuolar (*TaVI*, invertase genes expressed in the vacuole), while one belonged to the cytoplasmic (*TaCI*, invertase genes expressed in the cytoplasm) gene family.

**Figure 5:**
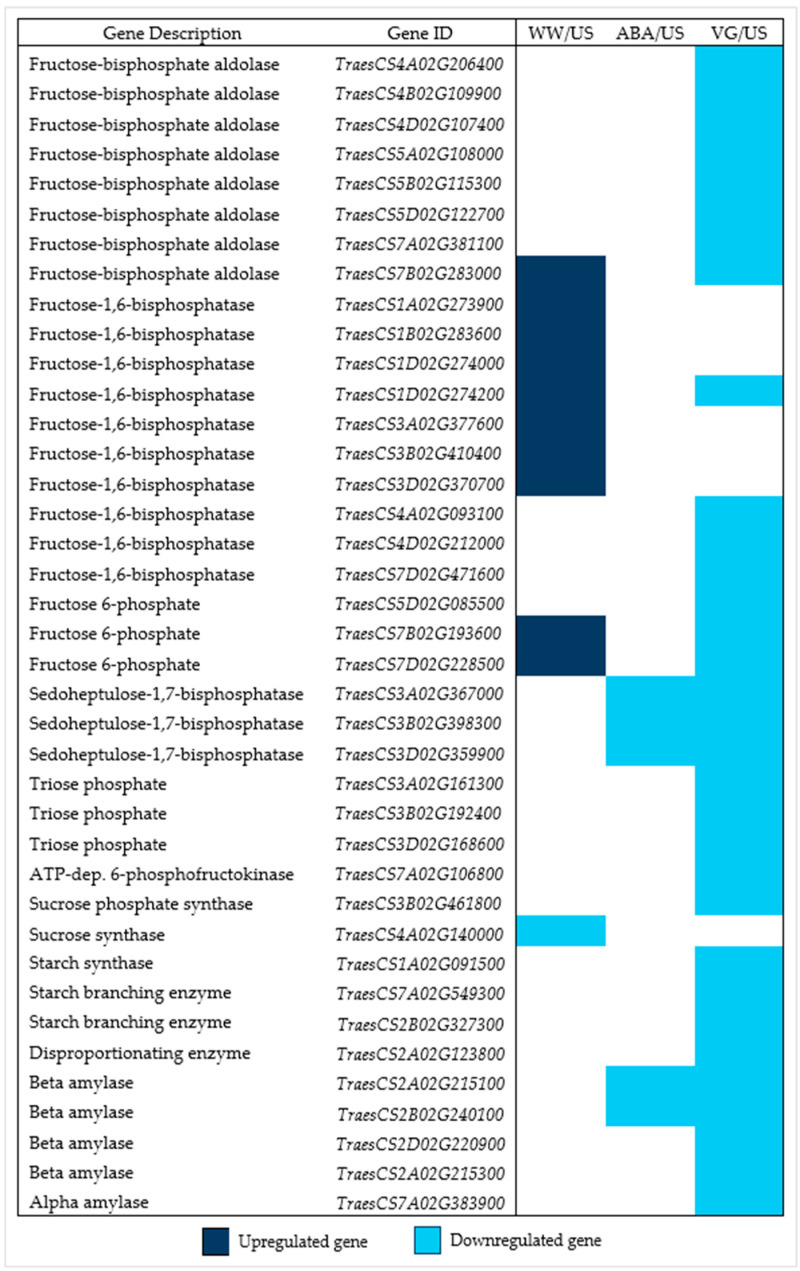
Sucrose biosynthesis genes and nine starch-related genes that were differentially expressed in different anther samples of three contrast comparisons (well-watered, ABA, and VG (droughted)-treated plant anthers compared to unsprayed ones). Dark blue represents upregulated genes, whereas light blue represents downregulated genes (padj < 0.05, log2 fold change > 1 (upregulated genes), and <−1 (downregulated genes)).

Some sucrose synthesis and starch-related genes, which were expressed in different anther samples, showed downregulation in droughted, treated plant anther samples, with most of them differentially expressed in VG anther samples than ABA anther samples (Figure 5). Some of the genes involved in sucrose synthesis (such as fructose-bisphosphate aldolase, fructose-1,6-bisphosphatase, and fructose 6-phosphate genes) showed upregulation in well-watered anthers, with either no or differential downregulation in treated samples; while nine starch-related genes (starch synthase, starch branching enzymes, disproportionating enzyme, and alpha and beta amylase genes) were not differentially expressed in well-watered anthers but only in antitranspirant-treated samples.

### 3.5. Differential Responses of Some Drought Tolerance and Response Genes

Two different types of ABA receptor genes were upregulated in ABA and VG anthers (*TraesCS1D02G195300* and *TraesCS2A02G089400*, respectively). Some transcription factors showed upregulation in well-watered anthers with no differential expression in treated samples (such as zinc finger protein—ZAT9 (C2H2), transcription factor ILI5 (bHLH), and WRKY28 genes) (Table S6). A few NAC and heat stress (HSF) transcription factors were upregulated in both antitranspirant anther samples, whereas some transcription factors were upregulated in VG samples only, belonging to zinc finger protein—ZAT12 (C2H2), MYB102, trihelix (GT-2), NAC, HD-ZIP, WRKY, and ERF gene families. Downregulated transcription factors mainly belong to bHLH, bZIP, BES1, MYB, and GRAS gene families, with differential expression either in ABA or VG samples. Protein phosphatase (PP2C) genes showed upregulation in VG anthers with downregulation of some of the protein kinase RIPK genes, ferritin, expansin, and inositol oxygenase genes (Table S6).

### 3.6. Pollen Viability and Yield

Pollen viability was around 92% in the well-watered plants, with an approximately 12% decrease in viability of droughted plants and no significant effect of antitranspirants on the pollen viability of different droughted treatments (Table S7). Similarly, yield per hectare was decreased by 35% under drought stress in comparison to the well-watered plants plots, while there was no significant difference between the yield of unsprayed and antitranspirant-treated plots and also no effect on other yield components was observed (Table S7).

## 4. Discussion

### 4.1. Antitranspirants Application Induces Major Transcriptomic Changes

Many differentially expressed genes have been identified or investigated in different crop anthers to understand their molecular mechanism under drought conditions, such as in wheat [16,19], rice [17], and tomato [43]. After performing pairwise DESeq2 analysis on three types of contrast comparisons (WW/US, ABA/US, and VG/US), hundreds to thousands of differentially expressed genes were found in different anther samples in the present study. In general, most of the genes in droughted anthers showed significant downregulation patterns in contrast to well-watered ones that were mostly upregulated. This altered and significant expression pattern of genes in different crop anthers was also summarised in a review paper [15], according to which drought is responsible for changes in crop pollen development that altered the expression of genes involved in sugar transport, hormonal response, reactive oxygen species, and meiotic process-related genes. This altered expression might help plants or anthers in repairing or avoiding any drought damage and protect their development in stressful environments. Consistent with previous studies, we found that drought stress affected carbohydrate/sugar metabolism and their transport, hormones, pollen/anther-related genes, and genes involved in cell cycle or cell division. Moreover, spraying antitranspirants significantly altered the expression pattern of the genes involved in these processes, as most of the genes related to these processes were downregulated under drought stress following the use of antitranspirants compared to unsprayed droughted plant anthers.

The number of downregulated genes was highest in VG-treated plant anthers (3325 genes) in comparison to ABA anthers (634 genes), which might suggest that plants with VG antitranspirant treatment were more sensitive or prone to the highest changes at the gene expression level, with most of the biological processes showing downregulation under water stress. Most of the genes that showed differential expression in VG anthers did not express in ABA anthers or showed no differential expression of genes involved in various biological processes; this indicates the unique transcriptional response to two different types of antitranspirant treatments, which may be due to the different mechanisms by which the antitranspirants reduce stomatal conductance. VG works by forming a physical barrier on the plant leaf surface to reduce transpiration, while ABA acts metabolically by either partially or fully closing stomatal pores to decrease transpiration from plants [44]. One other reason for this could be that plant stems from which VG anthers were harvested and stored in the PEG-400 solution longer (between 1 to 9 days maximum) than ABA ones (between 1 to 7 days maximum). Longer duration of storing in PEG-400 might have caused toxicity due to ion accumulation in the leaves, as one study [45] indicated increased cations (K^+^, Na^+^, Ca^2+^, and Mg^2+^) accumulation in the root xylem of pepper plants with PEG-400. However, such lower molecular weight PEGs are considered to have less effect on plant leaves in comparison to higher molecular weight PEGs [46].

### 4.2. Variations in Hormonal Responses for GO Enriched Terms

Hormonal balance in the reproductive parts of plants is very important, with abscisic acid (ABA), gibberellic acid, jasmonic acid, auxin, and cytokinin being the main ones responsible for reproductive development in plants and involved in drought tolerance mechanisms [15]. Drought stress significantly induces ABA biosynthesis genes in wheat anthers of drought-sensitive varieties, with lower ABA biosynthesis gene expression reported in drought-tolerant ones [47]. Spraying exogenous ABA under drought stress has been shown to increase flag leaf ABA levels, while VG spraying is suggested to be linked with low ABA levels of the same wheat variety (Chilham) [9] used for the present transcriptomic study. However, the present study indicated contrasting responses of ABA-related GO terms in VG and ABA anthers, with upregulation and downregulation of different genes involved in each case, respectively. This might be due to dissimilar mechanisms of how VG and ABA work and how they act on the endogenous ABA concentration differently at the physiological level [9], with contrasting transcriptomic responses of treated plant anthers for ABA hormone in this case.

Gibberellic acid (GA), another key plant hormone, plays an important role in rice anther development as it regulates the processes involved in exine formation and programmed cell death of tapetal cells [48]. A rice study indicated that GA-responsive genes were downregulated under drought stress at later anther developmental stages [17], whereas, in the early meiotic VG anthers, genes involved in the gibberellin biosynthesis and metabolic processes were upregulated as indicated via enriched GO terms, with no differential GO terms for these processes in ABA anthers. Genes related to jasmonic acid (JA) response are upregulated under abiotic stress conditions in many crop plants [49]; however, in this study, the JA response GO term (GO:0009753) was upregulated in VG anthers when compared to unsprayed anthers, with no enriched term in ABA anthers, indicating a distinct response of antitranspirants. Cytokinin (CK) hormone plays a key role in cell division processes of male and female reproductive parts of plants and is essential for anther or ovary development, which can be impacted under drought stress due to repression of CK signalling mechanism to adapt under stress conditions [50,51]. Present findings revealed that cytokinin-related genes were downregulated (as depicted by the response to cytokinin GO term GO:0009735, Table S3) with the spraying of VG antitranspirant compared to unsprayed plant anthers, but no differential expression was observed in other anther samples.

A previous study [17] revealed that genes related to cell wall development, microspore development, and starch synthesis are mostly affected by drought in rice florets, as observed in our study of anthers, while applying antitranspirants further repressed these genes compared to unsprayed plant anthers. However, pollen development GO term (GO:0009555, Table S3) showed contrasting results in ABA and VG anthers, with transcriptional downregulation and upregulation in each case, respectively. This suggests that although genes related to pollen wall formation and its composition products were affected by drought stress, some genes in VG anthers (related to pollen development) were transcriptionally higher in their expression, which might play a positive role in pollen viability. These genes might explain the mechanism of VG in improving pollen viability or crop yield, as suggested in a previous study [11].

### 4.3. Antitranspirants Affect Carbohydrate- or Sugar-Related Processes with Downregulation of Invertase, Starch, and Sucrose Synthesis Genes

Genes involved in carbohydrate or sugar metabolism in reproductive parts of plants are significantly affected under stressful environments and are the leading cause of poor pollen fertility and lower crop yield in different crop plants, e.g., rice [17,52], wheat [16,53], and tomato [43]. The enriched GO terms showed transcriptional downregulation of these processes in droughted, treated-plant anthers (VG and ABA) when compared with unsprayed, while in well-watered anthers, upregulation was observed. This suggests that antitranspirant spraying (either VG or ABA) did not ameliorate the effect of drought on carbohydrate or sugar metabolism because gene expression observed in well-watered plants was not restored in sprayed plants. It was also reflected in the pollen viability and crop yield results (Table S7), with antitranspirants showing no significant effect on plants compared to unsprayed ones, in contrast to previous studies of improved yield with antitranspirants [7,8,9,10,11,54,55].

Different studies revealed the importance of invertase genes in plants, as these are linked to carbohydrate or sugar metabolism and play a significant role in their development and stress tolerance [30,56]. These are involved in the conversion of sucrose into glucose and fructose, hormonal control mechanisms, and responses under stressful environments that affect seed or fruit sets [57]. Drought stress leads to changes in carbohydrate metabolism and a decline in invertase activity in wheat anthers, which affects the process of pollen development [19,58]. The invertase genes explored in this study revealed significant downregulation in treated anther samples compared to unsprayed ones (Figure 4), with no improved pollen viability or yield at the physiological level. This is contradictory to a wheat study [20], which found no change in expression of a vacuolar invertase (*IVR5*, referred to in Figure 4 as *TraesCS7D02G010000*) using a film antitranspirant. 

Accumulation of sugars and transient starch in the developing anthers serves as a vital source of energy, which is essential for the normal process of cell division and pollen maturation, determining their viability and germination. Any disturbance in the process of sugar utilisation and starch deposition in stressed anthers can cause pollen abortion [59,60]. Reproductive stage drought stress significantly reduces the expression of starch and sucrose synthesis genes in plant anthers, ultimately affecting pollen development and fertility due to the lack of necessary energy reserves [15,17]. Different types of enzymes, such as ADP-glucose pyrophosphorylase, starch synthase, starch branching, and debranching enzymes, are key for starch synthesis in crop plants and are associated with the conversion of sucrose to starch [61,62]. While beta and alpha amylases, along with disproportionating enzymes, are involved in starch degradation, they help plants in growth and development and respond to drought and other stresses. This breakdown of starch increases the soluble sugar content under drought, contributing to osmotic adjustments and helping plants to improve their tolerance to stress conditions [63,64,65]. In this study, most of the genes involved in starch or sucrose biosynthesis were affected under drought stress, and the application of antitranspirants further suppressed those genes. Consistent results of the downregulation of genes involved in starch or sucrose metabolism were observed due to drought stress from several other studies of crop plants [17,63,66,67]. However, one study [66] also observed that vacuole invertase and beta amylase genes were upregulated throughout drought stress during the jointing-booting stage in rice leaves. Whereas in this study, suppression of invertase and beta amylase genes was observed in VG-treated plant anthers compared to unsprayed ones under drought stress, which shows the opposite impact of antitranspirant spraying in the early meiotic anthers.

### 4.4. Antitranspirants Induce Variations in Expression Pattern of DEGs Involved in Drought Tolerance or Stress Response

Under drought stress, ABA regulates the expression of many target genes through ABA-responsive element (ABRE)-binding proteins/ABF (ABRE binding factor) transcription factors. ABA receptors (PYR/PYL/RCARs) perceive increased ABA concentration under drought stress, which leads to the inhibition of protein phosphatase 2C (PP2C). The released serine/threonine protein kinases (SnRK2s) regulate ABA-responsive gene expression by phosphorylating AREB/ABFs regulon genes [68,69]. These genes also regulate the expression of other drought response genes, including bZIP, MYB/MYC, DREB, WRKY, and NAC transcription factors, signalling protein kinases and protein phosphatases [68,70,71]. Differential expression of some of these genes was observed in droughted, treated-plants anther samples (ABA and VG), with varied gene expression responses compared to unsprayed samples (Table S6). Interestingly, some transcription factors were upregulated in VG anthers only (related to MYB102, C2H2-ZAT12, trihelix-GT2, phytochrome interacting factor 13 (bHLH), and homeobox-leucine zipper protein (HD-ZIP)), with no differential expression in other anther samples. Similar upregulation of homeobox-leucine zipper protein genes was observed in wheat plant samples collected at the pistil and stamen differentiation stage under drought stress conditions in a study [14], which suggests that in this study, VG spraying further upregulates these genes in anthers and thus may help plants under drought stress. Moreover, a few TFs (related to bHLH, bZIP, GRAS, and MYB families) showed the downregulation pattern in VG anthers, as also observed in rice florets at different developmental stages under drought stress [17] (with the same wheat orthologs of this study, Table S6). Some of the contrasting results in comparison to the previous rice florets study [17] were the upregulation of heat shock factors (HSF) in treated-plant anther samples, which might be due to the application of antitranspirants. Also, serine/threonine-protein kinase RIPK genes showed the downregulation in VG anthers in contrast to the upregulation of the same orthologs of these genes in rice florets under drought stress [17], which might have impacted the expression of other drought response genes.

## 5. Conclusions

Overall, this study shows that wheat plants are sensitive (especially male reproductive parts) to drought stress at the transcriptomic level, and their gene expression patterns are altered accordingly in a stressed environment. Applying antitranspirants can significantly change the transcriptomic responses in the early meiotic anthers. However, these transcriptomic changes might not lead to significant differences at the physiological level, such as in terms of crop yield, as observed in this study. Furthermore, the number and the type of genes and their expression patterns vary depending on the type of antitranspirant used. More research is required to fully understand how drought tolerance mechanisms work in different types of antitranspirant treatments and if applied at different growth stages of the crop. Target genes can then be identified and manipulated accordingly based on their transcriptional expression patterns under different stress environments and could be helpful for breeders in crop improvement or for creating knockouts to understand the gene of interest for a specific trait.

## Figures and Tables

**Table 1:** Wheat stems (main stem or primary tillers, with 3–4 leaves) sampling period (with dates and days’ range at which stems were collected and stored in refrigerator either in water or PEG solution) at the early meiosis stage (GS41) from well-watered (WW), droughted unsprayed (US), and droughted ABA and VG treated plant plots from the polytunnel, along with date of anthers collection or extraction from spikes at the leptotene-zygotene stage of meiosis I. The antitranspirants were sprayed on 4 August 2022 at the flag leaf stage (GS39). The unique name of the samples shows separate days when the sample was collected from the other replicate of that specific treatment.

Sample No.	Sample Name	Replicate No.	Date Range, Wheat Stems with 3–4 Leaves Were Collected and Placed in Water or PEG	Days’ Range, Stems with Leaves Were Stored in Water or PEG	Anthers Collection Date
1	WW1	1	2 August	1 day	3 August
2	WW2	2	2–3 August	1–2 days	4 August
3	WW4	3	2–7 August	1–6 days	8 August
4	US1	1	15 August	0 day	15 August
5	US3	2	15–16 August	1–2 days	17 August
6	US4	3	15–17 August	1–3 days	18 August
7	ABA2	1	15–21 August	1–7 days	22 August
8	ABA3	2	16–22 August	1–7 days	23 August
9	ABA5	3	17–23 August	1–7 days	24 August
10	VG1	1	16–24 August	1–9 days	25 August
11	VG2	2	16–24 August	1–9 days	25 August
12	VG5	3	17–25 August	1–9 days	26 August

## Data Availability

Data that supports the findings of this study is available in the Supplementary Information of this article. The raw RNA-seq reads are available from the European Nucleotide Archive with project number PRJEB90842. The scripts used for RNA-seq analysis are available from https://github.com/Borrill-Lab/Anther_Drought.

## Supplementary Materials

The following supporting information can be downloaded at: https://www.mdpi.com/article/10.3390/plants14172633/s1, Figure S1: Meteorological measurements. (**a**) Mean daily temperature with maximum and minimum temperature values recorded each day inside the polytunnels; (**b**) Mean daily relative humidity recorded inside the polytunnels during the cropping period; (**c**) Daily solar radiation data was taken from Harper Adams meteorological station for the cropping period which was located one km away from the experimental field site; Table S1: Total number of reads and pseudoaligned reads per sample; Table S2: Number of differentially expressed genes (upregulated and downregulated) in each contrast comparison with padj < 0.05 and log2fold change > 1 (for upreg.) and <−1 (for downreg.) values from DESeq2 analysis. Details of DEGs of each contrast are given on the next three separate sheets; Table S3: Enriched GO terms in three types of contrast comparison for differentially expressed genes (upregulated—dark blue and downregulated—light blue) with padj < 0.05. GO terms in each contrast are placed in order of highly significant at the top to least significant at the end of each contrast list. Also, the number of differentially expressed genes for each GO term in a particular category is listed with the number of genes in that GO term category in each contrast comparison; Table S4: Some of the selected enriched GO terms placed in a few main types of categories (hormones, proteins, carbohydrates, lipids, cell cycle terms, pollen, antioxidants, nutrients, and general terms related to stress) to understand the expression pattern of differentially expressed genes (upregulated—dark blue, downregulated—light blue and light yellow for terms appeared in both upregulated and downregulated genes) in three types of contrast comparisons; Table S5: Upregulated DEGs for pollen development GO term (GO:0009555) that came up after GO analysis in VG sprayed plant anther samples when compared with unsprayed. There were 34 DEGs that were linked to this pollen development GO term, given with their rice orthologs. Not all genes have rice orthologs; therefore, “N/A” is written to represent that in the table. Also, some wheat genes have more than one rice ortholog, as obtained after using the BioMart tool on the EnsemblPlants website. Table S6: Differential expression of some drought tolerance/response genes in different anther samples with candidate genes taken from various literature studies or EnsemblPlants/wGRN database; Table S7: Mean values of pollen viability and yield components (spike density per m^2^, number of grains per spike, thousand grain weight, number of grains per m^2^, and grain yield) of the 2022 field experiment in polytunnels, along with results (*p*-values with degree of freedom (df)) from the ANOVA analysis.
