# Supplementary material for: Transcriptomic Responses of Wheat Anthers to Drought Stress and Antitranspirants
Source: Plants (Basel). 2025 Aug 24;14(17):2633. doi: 10.3390/plants14172633 (PMC12430781; doi:10.3390/plants14172633)
Supplement: Supplementary file 1 [file plants-14-02633-s001.zip › Figure_S1.pdf]

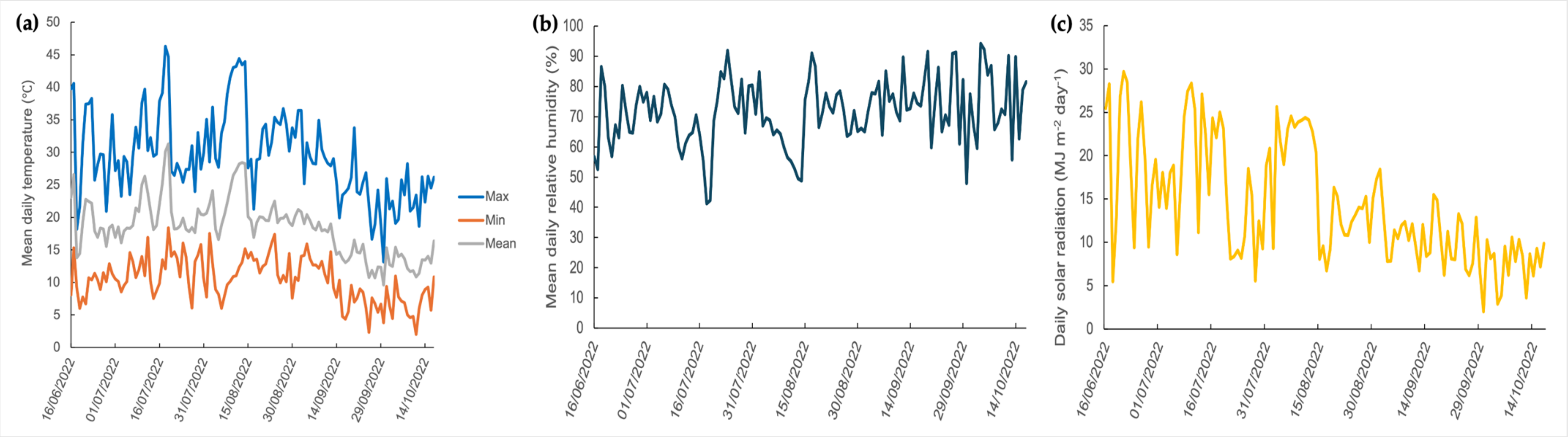

Figure S1: Meteorological measurements. (**a**) Mean daily temperature with maximum and minimum temperature values recorded each day inside the polytunnels; (**b**) Mean daily relative humidity recorded inside the polytunnels during the cropping period; (**c**) Daily solar radiation data was taken from Harper Adams meteorological station for the cropping period which was located one km away from the experimental field site.
