# Supplementary material for: Transcriptomic Responses of Wheat Anthers to Drought Stress and Antitranspirants
Source: Plants (Basel). 2025 Aug 24;14(17):2633. doi: 10.3390/plants14172633 (PMC12430781; doi:10.3390/plants14172633)
Supplement: Supplementary file 1 [file plants-14-02633-s001.zip › Table_S7.pdf]

Table S7: Mean values of pollen viability and yield components (spike density per m^2, number of grains per spike, thousand grain weight, number of grains per m^2 and grain yield) of the 2022 field experiment in polytunnels, along with results (p values with degree of freedom (df)) from the ANOVA analysis.

Treatment	Pollen viability (%)	Spike density per m^2	Number of grains per spike	Thousand grain weight	Number of grains per m^2	Grain yield (t/ha)
Well-watered	91.63	784.44	36.29	29.55	21932.86	6.51
Unsprayed droughted	82.70 (a)	562.50 (a)	26.32 (a)	35.78 (a)	12412.31 (a)	4.46 (a)
VG-GS39 droughted	78.33 (a)	500.83 (a)	26.02 (a)	34.43 (a)	10848.62 (a)	3.77 (a)
ABA-GS39 droughted	81.22 (a)	575.00 (a)	26.00 (a)	34.92 (a)	12467.05 (a)	4.35 (a)
p value (treatment) with (df)	0.197 (2)	0.111 (2)	0.980 (2)	0.143 (2)	0.195 (2)	0.142 (2)
SEM (residual df)	1.644 (14)	24.581 (13)	1.265 (13)	0.455 (13)	674.025 (13)	0.247 (13)

Well-watered values in all the parameters are given for comparison purposes only, as these were not included in the statistical analysis. Similar letters with mean values of each treatment indicate no significant differences between treatments from the Tukey post-hoc test. SEM represents the standard error of mean values.
